# AGE/RAGE axis regulates reversible transition to quiescent states of ALK-rearranged NSCLC and pancreatic cancer cells in monolayer cultures

**DOI:** 10.1038/s41598-022-14272-0

**Published:** 2022-06-14

**Authors:** Tetsuya Kadonosono, Kotaro Miyamoto, Shiori Sakai, Yoshiyuki Matsuo, Shojiro Kitajima, Qiannan Wang, Minori Endo, Mizuho Niibori, Takahiro Kuchimaru, Tomoyoshi Soga, Kiichi Hirota, Shinae Kizaka-Kondoh

**Affiliations:** 1 School of Life Science and Technology, Tokyo Institute of Technology, Yokohama, 226-8501 Japan; 2 Department of Human Stress Response Science, Institute of Biomedical Science, Kansai Medical University, Hirakata, 573-1010 Japan; 3 Institute for Advanced Biosciences, Keio University, Tsuruoka, 997-0052 Japan; 4 Center for Molecular Medicine, Jichi Medical University, Shimotsuke, 329-0498 Japan

**Keywords:** Cancer models, Lung cancer

## Abstract

Cancer recurrence due to tumor cell quiescence after therapy and long-term remission is associated with cancer-related death. Previous studies have used cell models that are unable to return to a proliferative state; thus, the transition between quiescent and proliferative states is not well understood. Here, we report monolayer cancer cell models wherein the human non-small cell lung carcinoma cell line H2228 and pancreatic cancer cell line AsPC-1 can be reversibly induced to a quiescent state under hypoxic and serum-starved (HSS) conditions. Transcriptome and metabolome dual-omics profiles of these cells were compared with those of the human lung adenocarcinoma cell line A549, which was unable to enter a quiescent state under HSS conditions. The quiescence-inducible cells had substantially lower intracellular pyruvate and ATP levels in the quiescent state than in the proliferative state, and their response to sudden demand for energy was dramatically reduced. Furthermore, in quiescence-inducible cells, the transition between quiescent and proliferative states of these cells was regulated by the balance between the proliferation-promoting Ras and Rap1 signaling and the suppressive AGE/RAGE signaling. These cell models elucidate the transition between quiescent and proliferative states, allowing the development of drug-screening systems for quiescent tumor cells.

## Introduction

Cellular quiescence is a dormant state of reversible cell cycle arrest. Quiescent tumor cells remain a major current challenge because they become resistant to cancer therapy, persist over prolonged periods of time by undergoing epigenetic modifications, adopt a new environment, elude immune control, and induce relapse and metastasis^1^. Therefore, quiescent tumor cells are a promising drug target for improving cancer outcomes^1–3^, and there is a high demand for appropriate in vitro models to study quiescent tumor cells. Cellular quiescence is mainly regulated by the extracellular matrix (ECM), cell signaling in the metastatic niche, and hypoxic and nutrient-reduced tumor microenvironment (TME)^3,4^. Owing to the recent advances in cellular quiescence biology and biomaterial, biofabrication, and microfluidic technologies, various in vitro quiescent tumor cell models are being developed^4,5^.

Several key factors involved in quiescent states have been reported using in vitro models associated with TME-mediated cellular quiescence. A study using sublines of human squamous cell carcinoma cell line HEp3, proliferative T-HEp3 and quiescent D-HEp3, identified the orphan nuclear receptor NR2F1 as a key regulator of TME-mediated cellular quiescence, and revealed that SOX9, RARβ, and cyclin-dependent kinase inhibitors, including p27 (CDKN1B), are involved in cellular quiescence^6,7^. Mouse embryonic fibroblasts and their gene-knockout mutants have contributed to the identification of the function of hypoxia-inducible gene domain family member 1A (HIGD1A) in TME-mediated cellular quiescence^8^. In these models, quiescent cells lose the ability to reenter the proliferative state; therefore, they are not suitable for elucidating the entire process involved in cellular quiescence.

A 3D-culture model that can mimic the oxygen and nutrient gradients of the TME is a desirable model for studying in vivo phenomena and has been used for the analysis of tumor cells in a TME-mediated quiescent state. The human colon cancer cell line HCT116 contains quiescent cells expressing p27 in the spheroid core region and proliferating cells in the peripheral region^9^. In this model, mitochondrial oxidative phosphorylation (OXPHOS) was found to be important for energy production in quiescent cells, and the mitochondrial inhibitor VLX600 showed strong cytotoxic activity against quiescent cells. Tumor cells in a quiescent state were also observed in the spheroid core of the human breast cancer cell lines BT-549 and BT-474^10^. Another 3D-culture model is cancer tissue-originated spheroids (CTOSs) from primary tumors. Proliferating cells in colorectal CTOSs enter a quiescent state with Akt suppression under hypoxic and growth factor-depleted conditions and re-proliferate under optimal culture conditions^11^. These 3D-culture models that allow the transition between quiescent and proliferative states may be more clinically relevant. However, the 3D-culture models have limitations in the analysis of the unique properties of quiescent cells. This is primarily because spheroids are composed of heterogeneous cell populations that require isolation of specific cell populations for analysis.

In the current study, we aimed to develop monolayer culture models capable of reversible transitions between quiescent and proliferative states to characterize recurrent tumor cells. Using the multiomics approach in the quiescence-inducible in vitro models, we identified the regulatory signaling axis that may determine the transition between the quiescent and proliferative states.

## Results

### H2228 and AsPC-1 cells were induced to a reversible quiescent state by hypoxia and low serum

**Figure 1:**
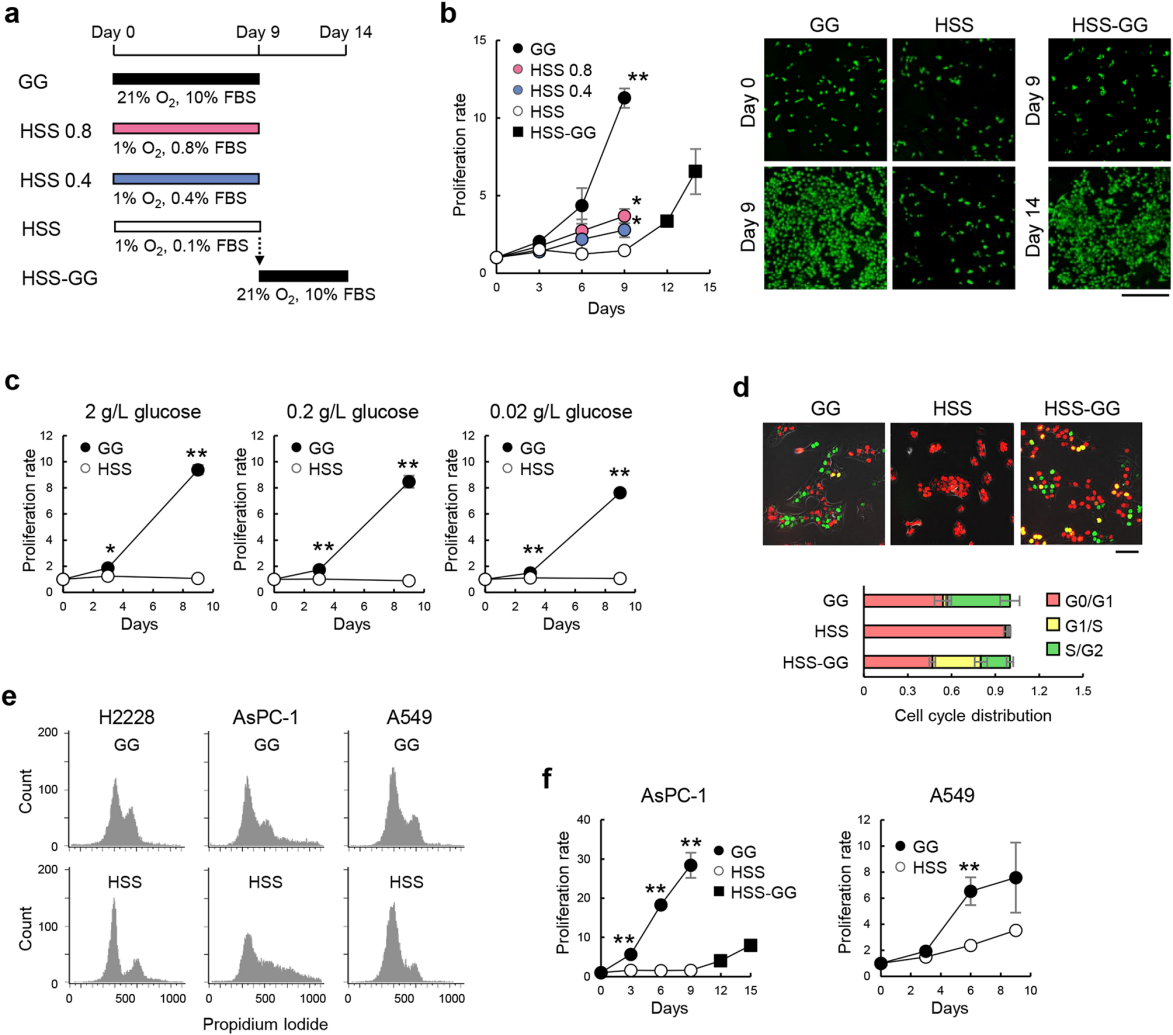
H2228 and AsPC-1 cells were induced to a reversible quiescent state. (**a**) Schematic representation of the cell culture conditions. (**b**) Proliferation of H2228 cells under GG and HSS conditions. Proliferation rates (left) and representative fluorescent micrographs (right) are shown. The living cells were fluorescently labeled with CellTracker Green CMFDA. (**c**) Proliferation of H2228 cells under GG and HSS conditions at different glucose concentrations. Proliferation rates are shown. (**d**) Cell cycle phase of H2228 under GG and HSS conditions. Representative fluorescent micrographs of H2228/fluorescent ubiquitination-based cell cycle indicator (FUCCI) cells (top) and cell cycle distribution (bottom) are shown. (**e**) Cell cycle analysis by quantitation of DNA content of H2228, AsPC-1, and A549 under GG and HSS conditions. Representative flowcytometric histograms after DNA staining with propidium iodide are shown. (**f**) Proliferation of AsPC-1 and A549 cells under GG and HSS conditions. Proliferation rates are shown. (**a, b, c, d, e, f**) GG, general growth conditions; HSS 0.8, hypoxic and serum-starved (1% O_2_ and 0.8% FBS) conditions; HSS 0.4, hypoxic and serum-starved (1% O_2_ and 0.4% FBS) conditions; HSS, hypoxic and serum-starved (1% O_2_ and 0.1% FBS) conditions; HSS-GG, GG conditions after HSS conditions; FBS, fetal bovine serum. **p < 0.01 (versus GG), *p < 0.05 (versus GG). Scale bar: 500 μm, *n* = 3.

To investigate the conditions under which tumor cells can be induced to a quiescent state in a few days, we used a human anaplastic lymphoma kinase (ALK)-rearranged non-small cell lung cancer (NSCLC) cell line H2228 because ALK-rearranged NSCLC patients treated with ALK tyrosine kinase inhibitors are known to have frequent cancer recurrence^12^. H2228 cells were cultured under hypoxic conditions in medium containing different concentrations of serum to induce a quiescent state for several days and returned to general growth (GG) conditions (21% O_2_, 10% fetal bovine serum [FBS]) to stimulate growth (Fig. 1a and b). Finally, we determined the conditions under which H2228 cells reversibly enter a quiescent state under hypoxic and serum-starved (HSS) conditions (1% O_2_, 0.1% FBS). The proliferation of H2228 cells was completely suppressed under HSS conditions within 3 days and restarted after changing the medium to GG (Fig. 1a and b). Surprisingly, glucose concentration did not influence the quiescence induction of H2228 cells (Fig. 1c). The experiment using H2228/fluorescent ubiquitination-based cell cycle indicator (FUCCI), the H2228 subline that stably carries FUCCI^13^, confirmed the reversible quiescence of H2228; H2228 cells were arrested in G0/G1 phases under HSS conditions within 3 days and entered the S phase 1 day after culturing under GG conditions (Fig. 1d). Cell cycle analysis by flow cytometry confirmed that the arrested H2228 was accumulated in the G0/G1 phases (Fig. 1e). These results demonstrate that HSS conditions are sufficient for H2228 cells to reversibly enter a quiescent state. To determine whether the induction of quiescence by HSS is applicable to other cell lines, we examined the human pancreatic cancer (PC) cell line AsPC-1, which has been reported to enter a quiescent state under prolonged hypoxia for 7 days^11^. HSS conditions are also sufficient for AsPC-1 to reversibly enter a quiescent state; AsPC-1 cell growth was arrested within 3 days under HSS conditions and started proliferating when the culture conditions were changed to GG on day 9 (Fig. 1f). AsPC-1 decreased G0/G1 cell population, and showed the presence of significant aneuploid cell populations under HSS conditions (Fig. 1e), suggesting that the regulation of the reversible transition may not simply correlate with G0/G1 arrest. The human lung adenocarcinoma cell line A549, which was reported to enter a quiescent state in an ECM-dependent manner^14^, had a reduced proliferation rate with the increased G0/G1 cell populations but failed to enter a quiescent state under HSS conditions (Fig. 1e and f). Therefore, we further examined the differences between these cell lines to elucidate the factors that determine reversible quiescence inducibility.

### ATP production by OXPHOS was limited in quiescence-inducible cells under HSS conditions

**Figure 2:**
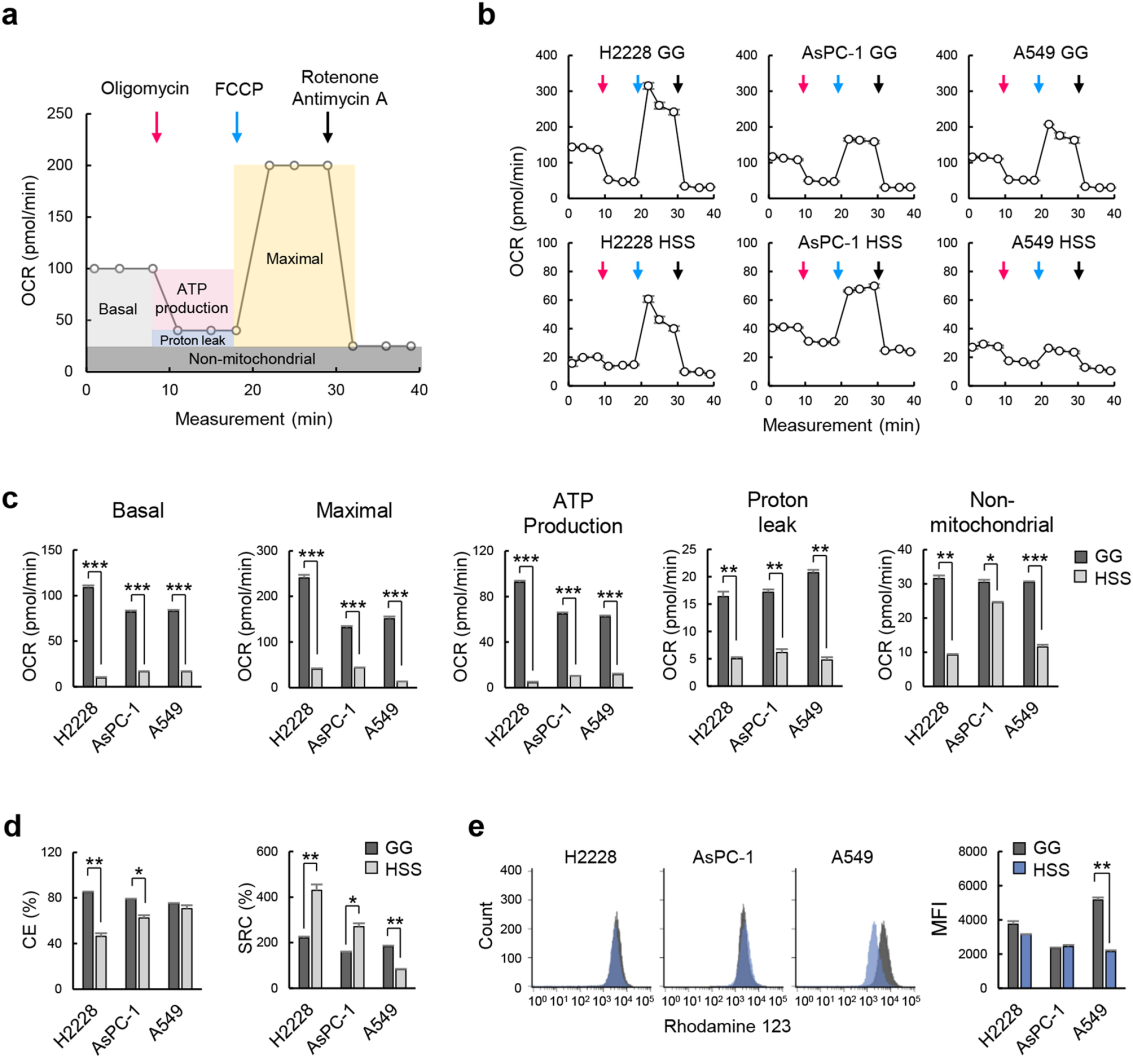
Quiescence-inducible cells generate small amounts of ATP by OXPHOS under HSS conditions. (**a**) Mitochondrial stress test profile of the key parameters of mitochondrial oxygen consumption rate (OCR). Basal, basal respiration; Maximal, maximal respiration; proton leak, proton leakage; non-mitochondrial, non-mitochondrial respiration. (**b**) OCR in H2228, A549, and AsPC-1 cells under GG and HSS conditions. Representative profiles are shown. Red, blue, and black arrows indicate the addition of oligomycin, FCCP, and rotenone and antimycin A, respectively. (**c**) The values of basal respiration (Basal), maximal respiration (Maximal), ATP production, proton leak, and non-mitochondrial respiration (Non-mitochondrial) from the profiles in (**b**). ***p < 0.001, **p < 0.01, *p < 0.05; *n* = 3. (**d**) The coupling efficiency (CE) and spare respiration capacity (SRC). CE was calculated as a ratio of ATP production and basal respiration. SRC was calculated as a ratio of maximal and basal respiration. **p < 0.01, *p < 0.05; *n* = 3. (**e**) Flow cytometric analysis of mitochondrial membrane potential of three cell lines under GG and HSS conditions. Representative histograms (left) and mean fluorescence intensity (MFI) values calculated from the histograms (right) are shown. **p < 0.01; *n* = 3.

OXPHOS is known to be important for energy production in quiescent cells^13^. To investigate the significance of OXPHOS in the quiescence-inducible cells, we analyzed the mitochondrial oxygen consumption rate of three cell lines using the mitochondrial stress test (Figs. 2a and b). The basal respiration, ATP production, and other parameters of oxygen-consuming processes (maximal respiration, proton leak, and non-mitochondrial respiration) of the three cell lines were remarkably lower under HSS conditions than under GG conditions (Fig. 2c). We then calculated the coupling efficiency (CE) and spare respiratory capacity (SRC), which represent the percentage of respiratory activity for ATP production and the extra mitochondrial capacity available in a case of sudden increase in energy demand, respectively. There were substantial differences between quiescence-inducible and quiescence-non-inducible cells; H2228 and AsPC-1 cells showed decreased CE and increased SRC under HSS conditions (Fig. 2d), indicating that respiratory activity did not increase in response to energy demand under HSS conditions. In contrast, as indicated by unchanged CE and reduced SRC, A549 cells used energy storage in response to energy demand to maintain normal respiratory activity and produce ATP even under HSS conditions (Fig. 2d). Energy storage through OXPHOS can be evaluated by measuring the mitochondrial membrane potential using rhodamine-123 fluorescent dye. The mitochondrial membrane potential was unchanged in H2228 and AsPC-1 cells but decreased in A549 cells under HSS conditions (Figs. 2e), confirming that A549 cells used energy storage under HSS conditions. These results suggest that an insufficient response to sudden demand for energy is one of the main determinants of reversible quiescence induction.

### Quiescence-inducible cells entered a quiescent state because of reduction in overall energy production under HSS conditions

**Figure 3:**
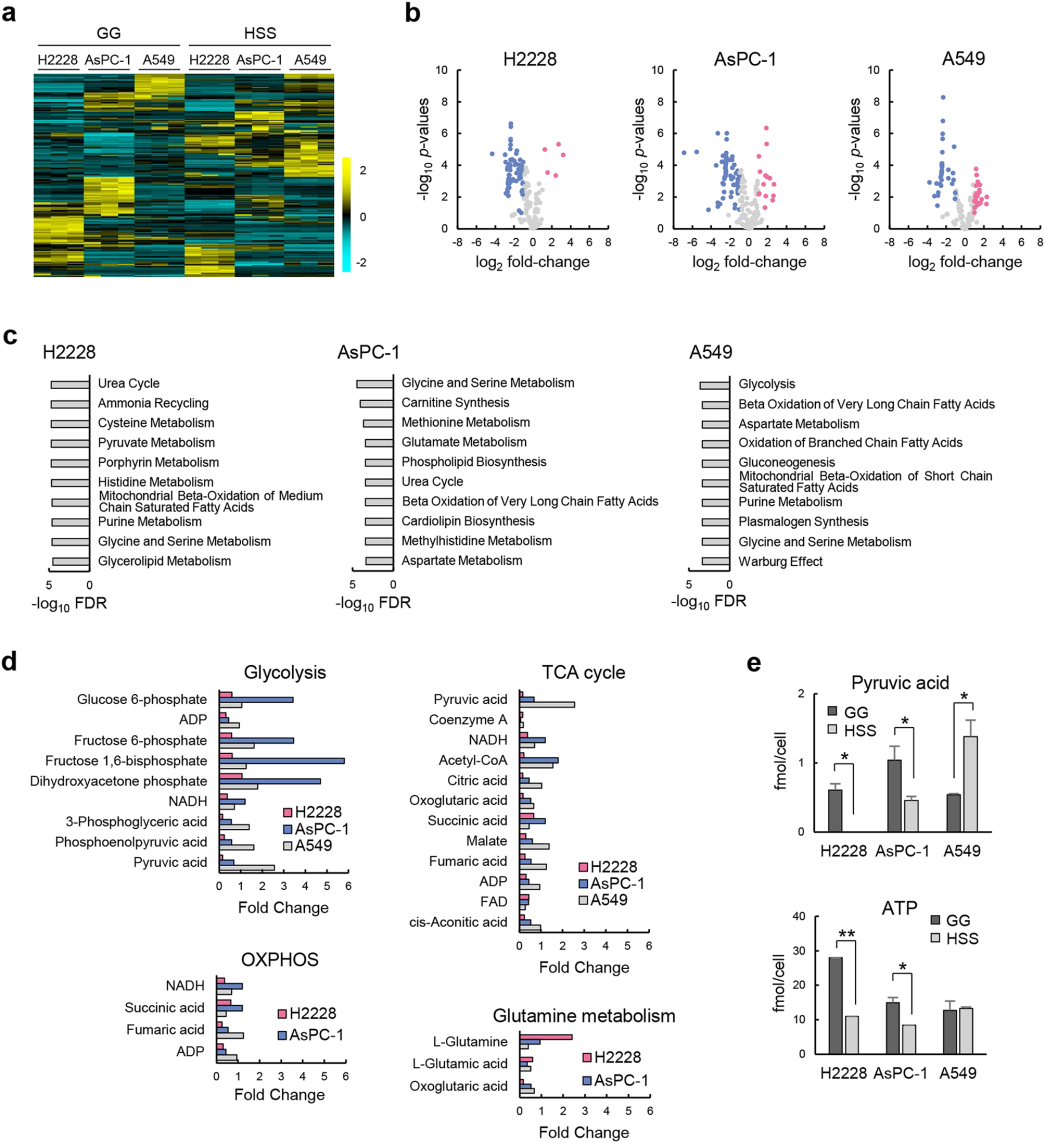
Overall energy metabolism was downregulated in quiescence-inducible cells under HSS conditions. (**a**) Heatmap showing metabolite concentration values in H2228, A549, and AsPC-1 cells under GG and HSS conditions; *n* = 3. Color scale (cyan to yellow) indicates relative metabolite concentration. (**b**) Volcano plots showing changes in metabolite concentrations in three cell lines under GG and HSS conditions; Increased and decreased metabolites under HSS versus GG conditions with a cut-off of *p* < 0.1 and fold-change > 2 are shown by red and blue plots, respectively. (**c**) Metabolite set enrichment analysis (MSEA) between cells under GG and HSS conditions. Altered metabolic pathways in each cell line were analyzed using the quantitative enrichment analysis (QEA) algorithm under HSS conditions compared to those under GG conditions. The ten most significantly altered metabolic pathways are shown. (**d**) Fold-change analysis of metabolite concentrations relating to energy metabolism. Fold-change of each metabolite in each cell line was calculated by dividing the mean concentration of each metabolite under HSS conditions by the mean concentration of each metabolite under GG conditions. (**e**) Concentration of pyruvic acid and ATP in three cell lines under GG and HSS conditions. **p < 0.01, *p < 0.05; *n* = 3.

To understand the differences in response to energy demand between quiescence-inducible and quiescence-non-inducible cell lines, the metabolite concentration values and a fold-change of each metabolite in three cell lines under HSS and GG conditions were analyzed and visualized with a heatmap and volcano plot, respectively (Fig. 3a and b). Metabolite set enrichment analysis (MSEA) revealed that metabolic pathways involved in amino acid, lipid, and nucleic acid metabolism were commonly enriched in the three cell lines (Fig. 3c). In contrast, the urea cycle and glycolysis were specifically enriched in the quiescence-inducible H2228 and AsPC-1 cells and the quiescence-non-inducible A549 cells, respectively (Fig. 3c). The fold-change of each metabolite related to energy metabolism under HSS versus GG conditions showed that the first half of glycolysis, up to dihydroxyacetone phosphate synthesis, was enhanced, but the second half of glycolysis and other metabolic pathways, tricarboxylic acid (TCA) cycle, OXPHOS, and glutamine metabolism tended to be suppressed in H2228 and AsPC-1 cells (Fig. 3d). As a result, pyruvic acid was depleted, and ATP was markedly reduced in H2228 and AsPC-1 cells (Fig. 3e). In the case of A549 cells, enhanced glycolysis increased pyruvate production and maintained intracellular ATP levels by compensating for a decrease in other pathways of ATP production under HSS conditions (Fig. 3d and e).

### HSS conditions suppressed cell cycle-related genes

**Figure 4:**
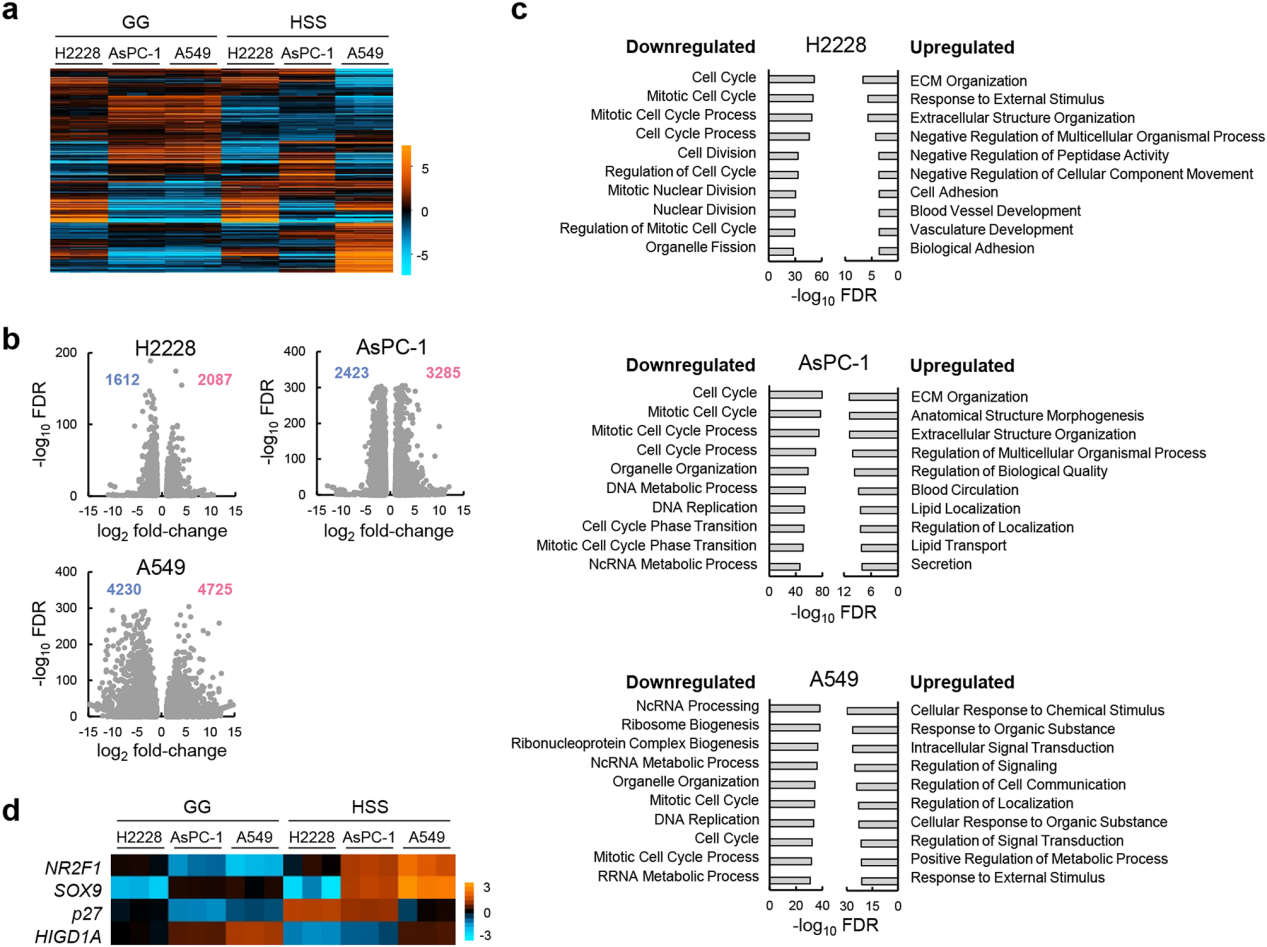
HSS conditions suppress cell cycle-related genes. (**a**) Heatmap showing the most variably expressed 1,000 genes among H2228, A549, and AsPC-1 cells under GG and HSS conditions; *n* = 3. Color scale (light blue to orange) indicates relative gene expression level. (**b**) Volcano plot showing differentially expressed genes (DEGs) in cells under GG and HSS conditions; Downregulated and upregulated genes under HSS conditions compared to GG conditions with a cut-off of false discovery rate (FDR) < 0.1 and fold-change > 2 are shown by red and blue plots, respectively. (**c**) Enrichment analysis for Gene Ontology (GO) biological process on DEGs from (b). The ten most downregulated and upregulated processes under HSS conditions compared to GG conditions in indicated cell line are shown. (**d**) Heatmap of relative expression levels of *NR2F1*, *SOX9*, *p27*, and *HIGD1A* genes in three cell lines under GG and HSS conditions. Color scale (light blue to orange) indicates relative gene expression level.

To elucidate gene expression patterns associated with reversible quiescence, gene expression profiles of quiescence-inducible (H2228 and AsPC-1) and quiescence-non-inducible (A549) cell lines cultured under GG and HSS conditions were analyzed by RNA sequencing. A heatmap showing the 1,000 most variable genes in the dataset had similar profiles between AsPC-1 and A549 cell lines under GG conditions but did not show a clear phenotype-related gene expression pattern (Fig. 4a). To identify differences in biological processes related to phenotype, differentially expressed genes (DEGs) that were increased or decreased under HSS conditions compared to under GG conditions (HSS vs GG) were extracted from each cell line (Fig. 4b), and enrichment analysis was performed using the Gene Ontology (GO) biological process (Fig. 4c). Among the 10 most significantly upregulated or downregulated processes, several processes related to the cell cycle were downregulated under HSS vs GG in all cell lines, confirming their growth-suppressing response to nutritional and oxygen deficiency (Fig. 1b and 1f). “ECM organization” and “extracellular structure organization” processes were commonly upregulated under HSS vs GG in H2228 and AsPC-1 cells, consistent with the recent study about dormancy and ECM remodeling^15,16^. Nonetheless, several processes involved in responses to external stimuli were upregulated under HSS vs GG in A549 cells, explaining their ability to grow continuously under HSS conditions (Fig. 1e). The results obtained thus far indicate that HSS conditions commonly suppress the cell cycle and main metabolic pathways and that the quiescence-inducible cells fail to activate alternative pathways to support proliferation and become quiescent, while A549 cells continue to grow by increasing their response to growth stimuli under HSS conditions.

Among *NR2F1*, *SOX9*, *p27*, and *HIGD1A* genes, which are known to be upregulated in the TME-mediated established quiescent cell lines^6–8^, only *p27* was upregulated in quiescence-inducible H2228 and AsPC-1 cells under HSS conditions (Fig. 4d). The other three genes were expressed differently regardless of the phenotype, indicating that these genes are not involved in reversible quiescence.

### Rap1 and Ras signaling may determine quiescence inducibility of H2228 and AsPC-1 cells

**Figure 5:**
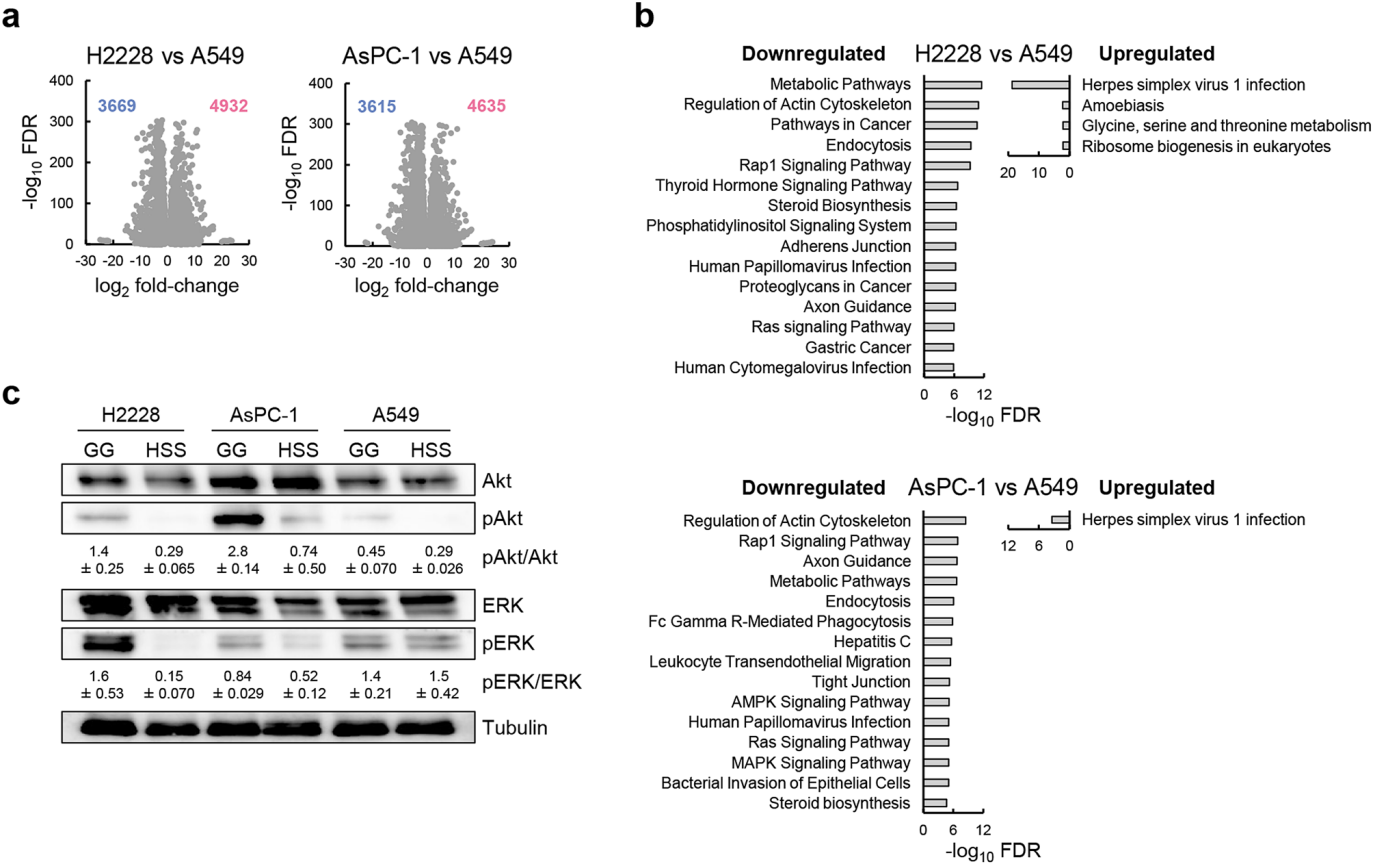
Akt and ERK phosphorylation were downregulated in quiescence-inducible cells under HSS conditions. (**a**) Volcano plot showing DEGs between H2228 or AsPC-1 versus A549 cells under HSS conditions; The number of upregulated and downregulated genes with a cut-off of FDR < 0.1 and fold-change > 2 is shown by red and blue, respectively. (**b**) Enrichment analysis against the Kyoto Encyclopedia of Genes and Genomes (KEGG) database on DEGs in (**a**). The 15 most significant downregulated pathways and all upregulated pathways are shown. (**c**) Immunoblots of total Akt, phosphorylated Akt, total ERK, phosphorylated ERK, and tubulin in H2228, A549, and AsPC-1 cells under GG and HSS conditions. The representative blot and relative signal intensities are shown; *n* = 3. Original blots are presented in Fig. S1.

To further investigate the unique responses in quiescence-inducible cells, we next analyzed DEGs between quiescence-inducible H2228 and AsPC-1 cells and quiescence-non-inducible A549 cells under HSS conditions (Fig. 5a). Enrichment analysis using Kyoto Encyclopedia of Genes and Genomes (KEGG) revealed that metabolic pathways, actin cytoskeleton dynamics, endocytosis, steroid biosynthesis, and several signaling pathways including Rap1 and Ras signaling pathways, were downregulated and herpes simplex virus 1 infection, which enhances central carbon metabolism toward the synthesis of pyrimidine nucleotides^17^, was upregulated in H2228 and AsPC-1 cells (Fig. 5b). Ablation of Ras signaling is known to generate dormant cell populations in pancreatic ductal adenocarcinoma models^18^. Thus, we measured the phosphorylation levels of Akt and ERK, downstream factors of Rap1 and Ras signaling. Immunoblotting showed that the phosphorylation of Akt and ERK was markedly decreased in H2228 and AsPC-1 cells under HSS conditions, whereas their phosphorylation was similar in A549 cells under GG and HSS conditions (Fig. 5c). These results suggest that a significant reduction in Rap1 and Ras signaling, which play an important role in H2228 and AsPC-1 cell proliferation under GG conditions, would be another determinant of reversible quiescence induction under HSS conditions.

### Aerobic glycolysis was elevated in quiescence-inducible cells under GG conditions

**Figure 6:**
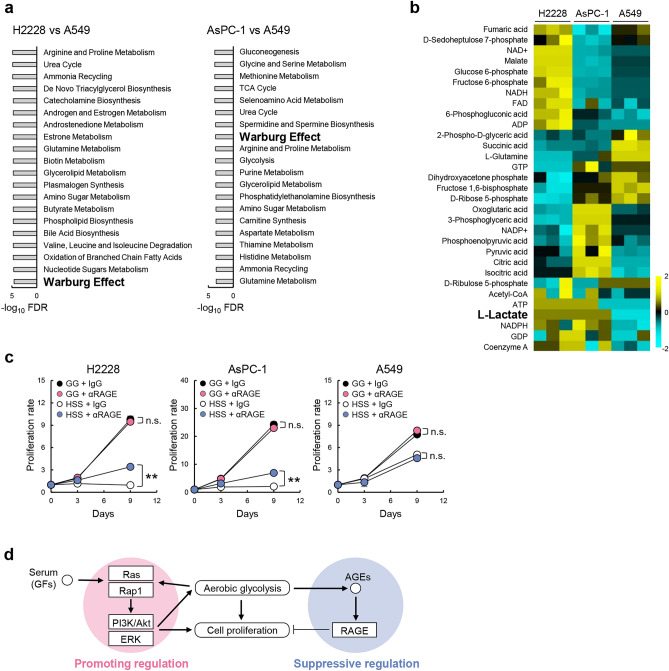
Aerobic glycolysis was elevated in quiescence-inducible H2228 and A549 cells under GG conditions. (**a**) MSEA between quiescence-inducible and quiescence-non-inducible A549 cell lines under GG conditions. Altered metabolic pathways were analyzed using the QEA algorithm. The 20 most significantly altered metabolic pathways are shown. (**b**) Heatmap showing relative abundance of metabolites relating to aerobic glycolysis among H2228, A549, and AsPC-1 cells under GG conditions; *n* = 3. Color scale (cyan to yellow) indicates relative metabolite concentration. (**c**) Proliferation of H2228, AsPC-1, and A549 cells under GG and HSS conditions with control IgG (IgG) or anti-receptor for advanced glycation end products (αRAGE) antibodies. Proliferation rates are shown. **p < 0.01. n.s., not significant; *n* = 3. (**d**) Proposed regulatory mechanism of cell proliferation in quiescence-inducible cells. GF, growth factor; AGE, advanced glycation end products.

Transcriptome analysis indicated that Ras and Rap1 signaling pathways were more significantly reduced in quiescence-inducible H2228 and AsPC-1 cells than in quiescence-non-inducible A549 cells under HSS conditions (Fig. 5b). Because glycolysis is enhanced by Ras signaling and Akt activation^19,20^ and stimulates Rap1 signaling^21^, aerobic glycolysis in quiescence-inducible cell lines may be high in H2228 and AsPC-1 cells under GG conditions. To verify this hypothesis, we analyzed the metabolic pathways of the three cell lines and compared the results of quiescence-inducible H2228 and AsPC-1 cells with those of quiescence-non-inducible A549 cells under GG conditions. The results of MSEA showed that the Warburg effect (aerobic glycolysis) was higher in H2228 and AsPC-1 cells (Fig. 6a). We then analyzed the relative abundance of metabolites related to aerobic glycolysis and found that L-lactate, the end product of aerobic glycolysis, was high in H2228 and AsPC-1 cells but not in A549 cells under GG conditions (Fig. 6b). Aerobic glycolysis induces the production of advanced glycation end products (AGE)^22^, which are known to suppress the proliferation of lung cancer cells by binding to the receptor for AGE (RAGE)^23^. To analyze the involvement of the AGE/RAGE signaling pathway in the quiescent phenotype, we blocked the pathway by adding a neutralizing antibody against RAGE into the culture medium and found that quiescence-inducible cells lost the ability to enter the quiescent state and continuously proliferated under HSS conditions (Fig. 6c). Based on these relationships of several pathways, we propose a regulatory mechanism of cell proliferation in the quiescence-inducible H2228 and AsPC-1 cell lines (Fig. 6d): serum components, including growth factors, promote cell proliferation and aerobic glycolysis through the activation of Ras, Rap1, PI3K/Akt, and ERK signaling, while AGEs produced by enhanced glycolysis suppress cell proliferation through the AGE/RAGE signaling pathway.

**Figure 7:**
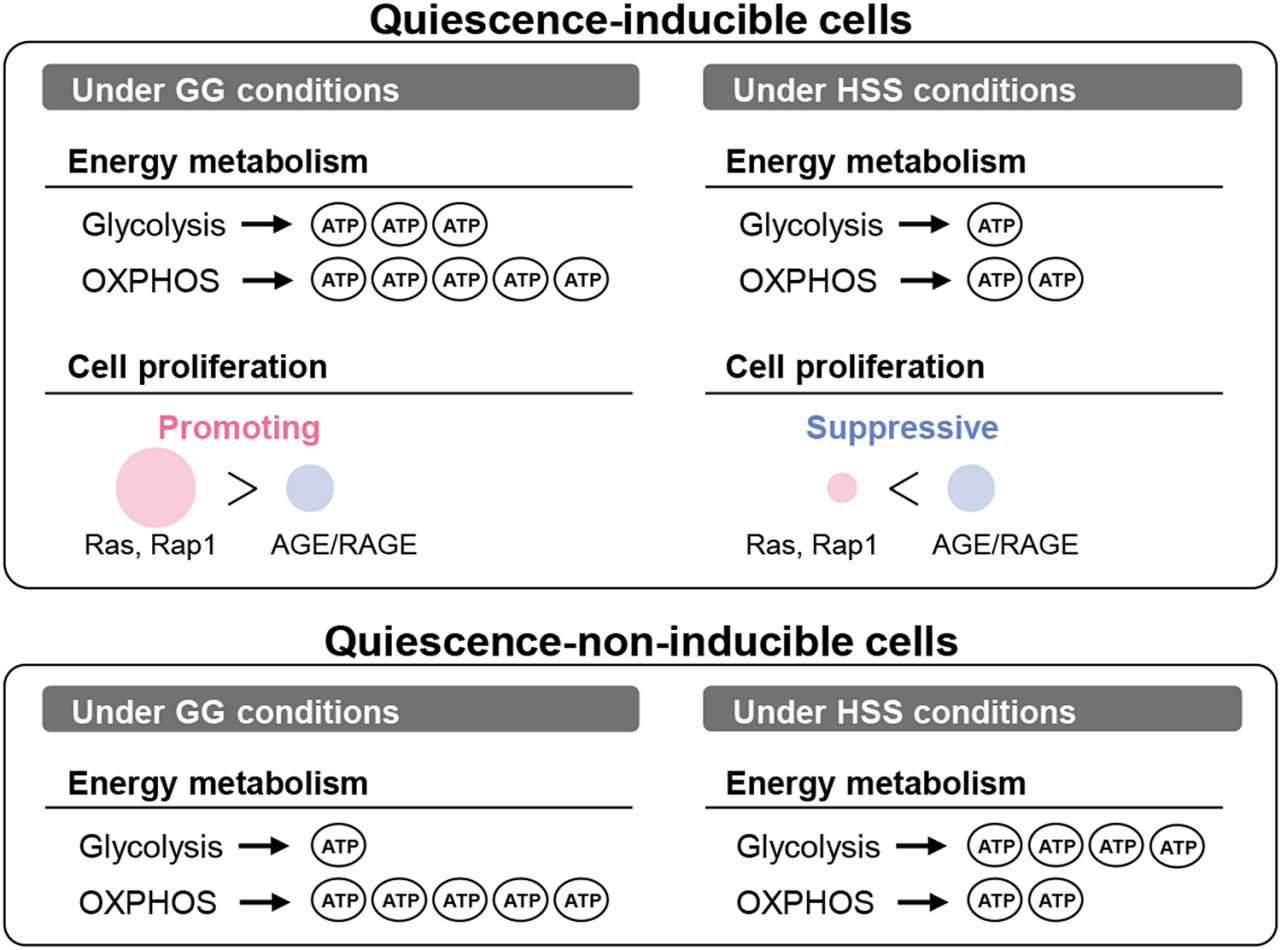
Schematic illustration of energy metabolism and regulation of cell proliferation. Both the quiescence-inducible and quiescence-non-inducible cells produce ATP by glycolysis and oxidative phosphorylation (OXPHOS) under GG conditions. Under HSS conditions, the quiescence-inducible H2228 and AsPC-1 cells produce a small amount of ATP mainly by OXPHOS and cell proliferation is suppressed through the advanced glycation end products (AGE)/receptor for AGE (RAGE) pathway, whereas the quiescence-non-inducible A549 cells activate glycolysis and produce a sufficient amount of ATP for cell proliferation.

These results indicate that quiescence-inducible cells produce ATP by enhanced aerobic glycolysis and OXPHOS, promoting cell proliferation through Ras and Rap1 pathways under GG conditions; the results further indicate that these processes were suppressed under HSS conditions, while cell proliferation was commonly suppressed through the AGE/RAGE pathway under GG and HSS conditions. In contrast, quiescence-non-inducible A549 cells remodeled ATP production systems from OXPHOS to glycolysis under HSS conditions to obtain sufficient energy to maintain cell proliferation (Fig. 7).

## Discussion

Quiescent tumor cells are promising drug targets for the prevention of tumor progression, metastasis, and recurrence. In the current study, we successfully developed monolayer culture models, which show the reversible transition between quiescent and proliferative states, using TME-relevant culture conditions. The biggest advantage of this model is that it is a monolayer culture that can be easily adapted to any analysis, including high-throughput drug-screening systems that contribute to drug development. More types of cancer cells need to be analyzed to verify whether the current findings are generalizable. However, these models are significant, at least, in suggesting the following: (i) cancer cells do not necessarily require gene mutations to exit a quiescent state; (ii) the control of energy metabolism during proliferation is also important for entering a reversible quiescent state; and (iii) the transition between quiescent and proliferating states is regulated by the balance between positive and negative signals activated in response to the TME.

We could not find significant relationship between ALK signaling pathway and quiescence transition by analyzing RNA sequencing data using GO Biological Process or KEGG database. H2228 has the *EML4-ALK* fusion gene but A549 is *EML4-ALK*-negative and expresses ALK at undetectable levels^24^. There is no report about ALK gene status of AsPC-1, but TAE684, a well‑known inhibitor of ALK, suppressed the proliferation of AsPC-1^25^, indicating that ALK signaling pathway contributes to AsPC-1 proliferation. ALK mutations activate many different pathways involved in cell proliferation and survival, including Ras/Raf/MEK/ERK1/2, JAK/STAT, PI3K/Akt, and PLC-γ pathways^12^. Therefore, ALK may be involved in the regulation of quiescence transition. Further study using cancer cell lines with *ALK* mutations will reveal the involvement.

The TME is characterized by hypoxia and low nutrition, which develop due to the high demand for oxygen and nutrients by highly proliferative cancer cells and aberrant angiogenesis^26^, and is thought to foster quiescent tumor cells^1–3^. Similar to many studies using TME-mediated quiescence models, we examined H2228 cells cultured with different concentrations of serum and glucose under hypoxic conditions. Serum concentration affected the quiescent induction of H2228, indicating that serum factor-induced growth signals are important for proliferation. In contrast, glucose concentration had no apparent effect on the induction of quiescence in H2228 cells. Since pyruvate concentration was so low that it could hardly be detected in H2228 cells under HSS conditions (Fig. 3e), glycolytic metabolism appears to be shut down regardless of the presence of glucose under HSS conditions. Further analysis is required to clarify the mechanism of this shutdown. Instead of glycolysis, H2228 cells may produce ATP through the TCA cycle using glutamine under HSS conditions. These metabolic characteristics are dramatically different from that of A549 cells, which is the same NSCLC cell type but does not enter the quiescent state under HSS conditions. A549 cells showed enhanced glycolytic metabolism to maintain the amount of ATP (Fig. 3e), exhibiting a typical hypoxic adaptive metabolic shift under HSS conditions.

A previous study using ECM-mediated quiescent tumor cell models found that the transition between quiescent and proliferative states was dependent on fibronectin production and signaling through integrin β1, leading to cytoskeletal reorganization with filamentous actin stress fiber formation^27^. In our model, the TME-relevant quiescence-inducible cells showed increased ECM organization and extracellular structure organization (Fig. 4c), which are processes for generating ECM, but decreased actin cytoskeleton dynamics under HSS conditions (Fig. 5b). These results suggest that TME-relevant quiescent cells have different quiescent properties from ECM-mediated quiescent cells. One possible explanation is the difference in aerobic glycolysis, which is significantly reduced in our model under HSS conditions, because glycolysis is reported as a primary bioenergetic pathway for cell motility and cytoskeletal remodeling^25^.

The 3D-cultured spheroid models of human mammary epithelial cell line MCF-10A show an increase in the urea cycle in quiescent cells, providing evidence for changes in nitrogen metabolism between quiescent and proliferative cells^28^. As a result, the amount of cellular amino acids decreased and the top hit for pathway topology was alanine, aspartate, and glutamate metabolism in the quiescent cells^28^. Upregulation of the urea cycle and several amino acid metabolism pathways were also enhanced in our quiescence-inducible model under HSS conditions (Fig. 3c), indicating that the changes in nitrogen metabolism were similar to those in quiescent MCF-10A cells.

We observed enhanced aerobic glycolysis in quiescence-inducible cells under GG conditions (Fig. 6a and b) and proposed a Ras and Rap1 pathway-dependent activation of aerobic glycolysis (Fig. 6d). We also propose that the quiescent phenotype of quiescence-inducible cells is regulated by the balance between the proliferation-promoting Ras and Rap1 pathways and the suppressive AGE/RAGE pathway (Fig. 6d). AGE/RAGE-mediated cancer malignancy has been extensively studied in recent years^29^, including the enhancement of proliferation, migration, and invasion during breast and prostate cancer progression^30,31^. The potential involvement of AGE in colorectal carcinogenesis has been suggested by clinical observation^32^. Moreover, the interaction between AGE/RAGE and Ras is reported to induce the activation of HIF1α and enhance tumor aggressiveness^33^. Therefore, our proposed signaling pathway might be a general model for understanding malignant cancer cells.

In our study, we focused on reversible quiescence because we believe that the reversible transition between quiescent and proliferating states can be caused by changes in cell signaling, metabolism, epigenetics, or all of these, without genetic mutations, and that tumor cells capable of reversible quiescence have unique properties that are detectable even in a proliferative state. If these hypotheses are correct, it may be possible to more effectively detect cancer cells that have the potential to enter a quiescent state, and thus develop drugs that prevent malignant progression and recurrence. Lactate production and AGE/RAGE signaling pathways were elevated in quiescence-inducible H2228 and AsPC-1 cells under GG conditions compared to those in quiescence-non-inducible A549 cells. Further studies using other tumor cell lines and clinical samples are necessary to generalize the unique properties of quiescence-inducible tumor cells. These results, however, suggest that tumor cells that contribute to recurrence have common and unique properties that can be therapeutic targets.

Quiescent cells in the core region of the HCT116 multicellular spheroid (MCS) showed a dependency on OXPHOS for maintenance of cellular ATP levels, resulting in the identification of the mitochondrial inhibitor VLX600 using HCT116 MCS as a drug-screening system targeting quiescent cells^9^. Our model demonstrated that quiescence-inducible cells generated small amounts of ATP mainly by OXPHOS under HSS conditions, suggesting that our model can also be used as a screening system to identify drugs that target mitochondrial functions in quiescent tumor cells. Moreover, monolayer-cultured models are useful for studying quiescent tumor cells because they are a uniform cell population to which advanced techniques such as clustered regularly interspaced short palindromic repeats (CRISPR) gene screening can be applied. Therefore, our model may reveal a more detailed mechanism of action of drugs than MCS models.

In conclusion, our study describes the successful development of a monolayer-cultured, reversible, and TME-mediated quiescent cancer cell model, sharing several features relevant to quiescent tumor cells in vivo. The quiescence-inducible cells may provide a better system for advanced in vitro analysis at the single-cell level and for drug screening of quiescent tumor cells.

## Materials and methods

### Ethics statement

All recombinant DNA experiments were performed with the approval of the recombinant DNA advisory committees of the Tokyo Institute of Technology. All methods were performed in accordance with relevant guidelines and regulations.

### Cell lines

The H2228 and A549 human lung and AsPC-1 human pancreatic cancer cell lines were purchased from the American Type Culture Collection (ATCC, Manassas, VA, USA). H2228/FUCCI was established after lentiviral transduction of the gene encoding mKO2-hCdt1(30/120) and mAG-hGeminin(1/110) in pFucci-G_1_ Orange and pFucci-S/G_2_/M Green-Hyg vectors (MBL, Aichi, Japan), respectively, into H2228 cells. H2228, H2228/FUCCI, and AsPC-1 or A549 cells were maintained in a 5% CO_2_ incubator at 37 °C with 10% FBS-RPMI1640 (Thermo Fisher Scientific, Waltham, MA, USA) or 5% FBS-DMEM (Thermo Fisher Scientific) media, respectively. Penicillin (100 U/ml) and streptomycin (100 µg/ml) (Nacalai Tesque, Kyoto, Japan) were added to all media. The cell lines were regularly checked for mycoplasma contamination using a mycoplasma check kit (Lonza, Basel, Switzerland) and were independently stored and recovered from the original stock every time for each experiment.

### Cell proliferation assay

Cell culture under hypoxic and serum-starved conditions was achieved by incubating the cells in a multigas incubator (ASTEC, Fukuoka, Japan). H2228, AsPC-1, and A549 (1.0 × 10^3^ cells/well) were seeded in a 96-well plate (Thermo Fisher Scientific) and cultured under HSS (1% O_2_, 5% CO_2_, and 0.1% FBS-RPMI1640 or 0.1% FBS-DMEM) or GG (21% O_2_, 5% CO_2_, and 10% FBS-RPMI1640 or 5% FBS-DMEM) at 37 °C for 9 days. To analyze re-proliferation, the quiescent H2228 and AsPC-1 cells were additionally cultured after changing to GG conditions for 5 days. For analyzing the effects of FBS or glucose concentrations, the H2228 (1.0 × 10^3^ cells/well) were cultured under HSS (1% O_2_, 5% CO_2_, 0.1% FBS, and 2 g/L glucose-RPMI1640), HSS 0.4 (1% O_2_, 5% CO_2_, 0.4% FBS, and 2 g/L glucose-RPMI1640), HSS 0.8 (1% O_2_, 5% CO_2_, 0.8% FBS, and 2 g/L glucose-RPMI1640), HSS 0.2G (1% O_2_, 5% CO_2_, 0.1% FBS, and 0.2 g/L glucose-RPMI1640), HSS 0.02G (1% O_2_, 5% CO_2_, 0.1% FBS, and 0.02 g/L glucose-RPMI1640), GG (21% O_2_, 5% CO_2_, 10% FBS, and 2 g/L glucose-RPMI1640), GG 0.2G (21% O_2_, 5% CO_2_, 10% FBS, and 0.2 g/L glucose-RPMI1640), or GG 0.02G (21% O_2_, 5% CO_2_, 10% FBS, and 0.02 g/L glucose-RPMI1640) conditions at 37 °C for 9 days. To analyze the effect of AGE/RAGE signaling, H2228, AsPC-1, and A549 (1.0 × 10^3^ cells/well) were cultured under HSS or GG conditions with 1 μg/100 μL mouse IgG2B isotype control (R&D Systems, Minneapolis, MN, USA) or mouse anti-human RAGE neutralizing antibody (R&D Systems) at 37 °C for 9 days. The living cells were fluorescently labeled with CellTracker Green CMFDA (Thermo Fisher Scientific) and counted using a BZ-X700 microscope (Keyence, Osaka, Japan) with an appropriate filter (Ex/Em = 470 ± 40 nm/520 ± 50 nm) on the indicated days. The proliferation rate was calculated as the ratio of the cell number at the indicated days compared with the cell number on day 0.

### Cell cycle analysis

H2228/FUCCI (1.0 × 10^6^ cells) were cultured in 10-cm dishes under HSS or GG conditions at 37 °C for 3 days. To analyze re-proliferation, quiescent H2228/FUCCI cells were further cultured after changing to GG conditions for 1 d. The cell cycle phase of H2228/FUCCI was analyzed using a BZ-X700 microscope with the appropriate filters (Ex/Em = 470 ± 40 nm/520 ± 50 nm for mAG and Ex/Em = 545 ± 25 nm/605 ± 70 nm for mKO2). The number of mAG( +)/mKO2(-) (green), mAG( +)/mKO2( +) (yellow), and mAG(-)/mKO2( +) (red) cells in the micrographs were counted as the cells in S/G2, G1/S, and G0/G1 phases, respectively, and the cell cycle distribution was calculated as the cell number of each cell cycle phase compared to the total cell number.

As for the flowcytometric cell cycle analysis by quantitation of DNA content, H2228, AsPC-1, and A549 cells (1.0 × 10^5^ cells) were cultured in 10-cm dishes under HSS or GG conditions at 37 °C for 5 days. The cells were harvested, gently suspended in PBS and mixed with equal volume of 2 × hypotonic fluorochrome solution (100 μg/ml propidium iodide in 0.2% sodium citrate and 0.2% Triton X-100) immediately before the analysis with a flow cytometry using a flow cytometer iCyt ec800 (Sony Biotechnology, CA, USA).

### RNA-seq analysis

H2228, AsPC-1, and A549 cells were cultured in 10-cm dishes under HSS or GG conditions at 37 °C for 3 days. Total RNA was purified from all the cultured cells (1.0 × 10^6^ cells) using the QIAGEN RNeasy Mini Kit (Qiagen, Valencia, CA, USA) according to the manufacturer’s instructions. Sequencing was performed on an Illumina NovaSeq 6000 platform by Macrogen Japan Corp. (Tokyo, Japan) with an average read depth of 52 million reads per sample. RNA-seq reads were quantified and analyzed using the iDEP.91^34^. A hierarchical clustering heatmap was generated using the Pearson correlation. DEGs were identified using DESeq2 (FDR < 0.1, fold change > 2) and used for fold change analysis and enrichment analysis with the gene set of GO Biological Process or KEGG^35,36^ database.

### Immunoblot analysis

H2228, AsPC-1, and A549 (1.0 × 10^5^ cells) were cultured in 10-cm dishes under HSS or GG conditions at 37 °C for 3 days and then lysed with Laemmli sample buffer. The protein samples were separated on 12.5% SDS–polyacrylamide gels and transferred to Hybond ECL membranes (GE Healthcare, Little Chalfont, UK). Akt, pAkt, ERK, pERK, and tubulin were detected with monoclonal anti-Akt (#2920), anti-phospho-Akt (#4060), anti-ERK 1/2 (#9102), anti-phospho-ERK 1/2 (#4377), and anti-α-tubulin antibody (#2144) (Cell Signaling Technology, Danvers, MA, USA), respectively. Appropriate horseradish peroxidase-conjugated secondary antibodies were obtained from Cell Signaling Technology.

### Metabolites analysis

H2228, AsPC-1, and A549 cells were cultured in 10-cm dishes under HSS or GG conditions at 37 °C for 3 days. Cells (1.0 × 10^6^ cells) were washed twice with ice-cold 5% mannitol solution and covered with 1 mL of methanol containing 25 μM internal standards, methionine sulfate (FUJIFILM Wako Pure Chemical, Osaka, Japan), 2-(N-morpholino)-ethanesulfonic acid (FUJIFILM Wako Pure Chemical), and D-camphor-10-sulfonic acid (FUJIFILM Wako Pure Chemical). The collected samples (400 μL) were mixed with 400 μL of chloroform and 200 μL of Milli-Q water. The aqueous phase of the cell sample was then subjected to ultrafiltration. Metabolites of cells were quantified using CE-MS (Agilent Technologies, Santa Clara, CA, USA) as previously described with automatic integration software (MasterHands version 2.17.3.18, Keio University)^37^ and analyzed using MetaboAnalyst 5.0^38^. A hierarchical clustering heatmap was generated using the Euclidean correlation. The fold change of each metabolite was measured by comparing the absolute value of change between the two groups. The small molecule pathway database (SMPDB) was used for metabolite set enrichment analysis (MSEA).

## Extracellular flux analysis

The H2228 (2.0 × 10^4^ cells), AsPC-1 (1.2 × 10^4^ cells), and A549 (1.5 × 10^4^ cells for HSS or 5.0 × 10^3^ cells for GG conditions) were seeded in the well of Seahorse XFp Cell Culture microplates (Agilent Technologies) and incubated under HSS conditions for 3 days or GG conditions overnight, respectively. The XF Cell Mito Stress Test™ was performed in glucose-containing XF base medium, following the manufacturer’s instruction with 1 µM oligomycin, 0.5–2 µM FCCP, 0.5 µM rotenone, and 0.5 µM antimycin A.

### Membrane potential analysis

H2228, AsPC-1, and A549 (1.0 × 10^5^ cells) were cultured in 10-cm dishes under HSS or GG conditions at 37 °C for 3 days. The cells were incubated with 1 µg/mL Rhodamine 123 (FUJIFILM Wako Pure Chemical) in the medium for 15 min and collected by trypsinization. Flow cytometric analysis was performed using a flow cytometer iCyt ec800 (Sony Biotechnology, CA, USA).

### Statistics

All experimental data were acquired three times and used for the statistical analysis. Data are presented as means ± standard error of the mean (SEM) and were statistically analyzed using a two-sided Student’s *t*-test; *p* values of < 0.05, were considered statistically significant.

## Supplementary Information

Supplementary Information.

## Data Availability

Generated RNA-seq data have been deposited in the DNA Data Bank of Japan (DDBJ) under the accession codes of Bio Project ID: PRJDB13075 and DRA submission ID: DRA013513. All data generated during this study are available from the corresponding author on reasonable request.
