# Supplementary material for: AGE/RAGE axis regulates reversible transition to quiescent states of ALK-rearranged NSCLC and pancreatic cancer cells in monolayer cultures
Source: Sci Rep. 2022 Jun 14;12:9886. doi: 10.1038/s41598-022-14272-0 (PMC9198021; doi:10.1038/s41598-022-14272-0)
Supplement: Supplementary file 1 — Supplementary Information. [file 41598_2022_14272_MOESM1_ESM.pdf]

Supplementary Information for

Title

AGE/RAGE axis regulates reversible transition between quiescent and proliferative states of NSCLC and pancreatic cancer cells in a monolayer model

Authors

Tetsuya Kadonosono, Kotaro Miyamoto, Shiori Sakai, Yoshiyuki Matsuo, Shojiro Kitajima, Qiannan Wang, Minori Endo, Mizuho Niibori, Takahiro Kuchimaru, Tomoyoshi Soga, Kiichi Hirota, and Shinae Kizaka-Kondoh

This PDF file includes:

Fig. S1

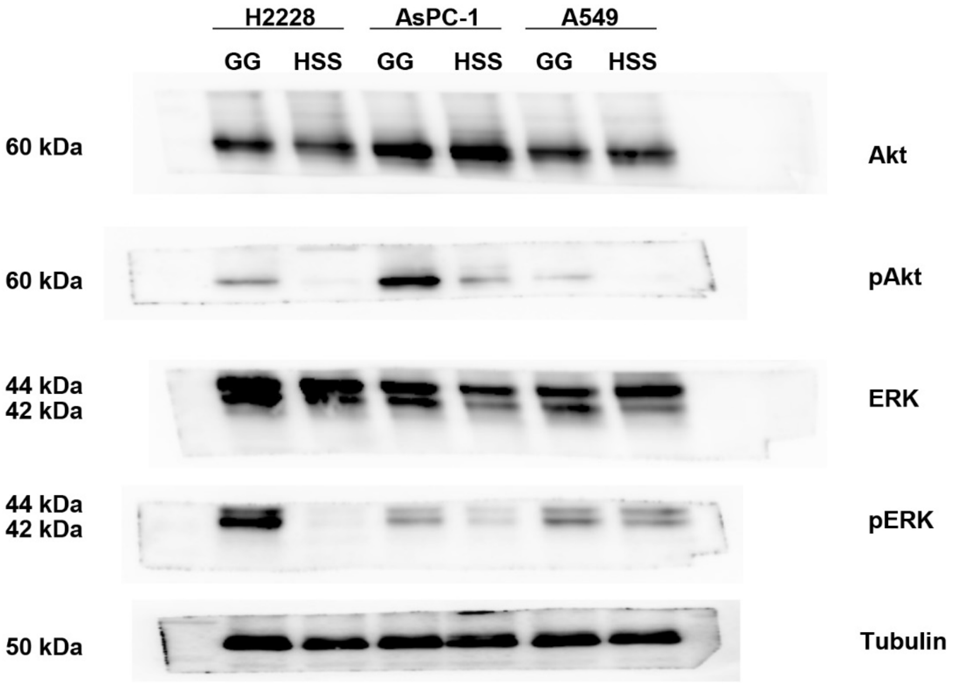

Figure S1. Full scans of original blots for data in Figure 5c.

Full-length immunoblots of Akt, pAkt, ERK, pERK, and Tubulin in Figure 5c. The membrane blots represented unprocessed original image data.
